# Tacrolimus CYP3A Single-Nucleotide Polymorphisms and Preformed T- and B-Cell Alloimmune Memory Improve Current Pretransplant Rejection-Risk Stratification in Kidney Transplantation

**DOI:** 10.3389/fimmu.2022.869554

**Published:** 2022-06-27

**Authors:** Elena Crespo, Anna Vidal-Alabró, Thomas Jouve, Pere Fontova, Maik Stein, Sonila Mocka, Maria Meneghini, Anett Sefrin, Petra Hruba, Montserrat Gomà, Alba Torija, Laura Donadeu, Alex Favà, Josep M. Cruzado, Edoardo Melilli, Francesc Moreso, Ondrej Viklicky, Frederike Bemelman, Petra Reinke, Josep Grinyó, Nuria Lloberas, Oriol Bestard

**Affiliations:** ^1^ Nephrology and Transplant Laboratory, Vall d'Hebron Institute of Research (VHIR), Barcelona, Spain; ^2^ Experimental Nephrology and Transplantation Laboratory, Instituto de Investigación Biomédica de Bellvitge (IDIBELL), Barcelona, Spain; ^3^ Faculty of Health, Université Grenoble Alpes, Grenoble, France; ^4^ Institute for Advanced Biosciences, INSERM 1209, CNRS 5309, Grenoble, France; ^5^ Berlin Center for Advanced Therapies (BeCAT), Berlin, Germany; ^6^ Charité - Universitätsmedizin Berlin, corporate member of Freie Universität Berlin, Humboldt-Universität zu Berlin, Berlin, Germany; ^7^ Berlin Institute of Health (BIH), Berlin, Germany; ^8^ Kidney Transplant Unit and Nephrology Department, Vall d’Hebron Hospital, Barcelona, Spain; ^9^ Department of Nephrology, Institute for Clinical and Experimental Medicine (IKEM), Prague, Czechia; ^10^ Pathology Department, Bellvitge University Hospital, Barcelona, Spain; ^11^ Kidney Transplant Unit, Nephrology Department, Bellvitge University Hospital, Barcelona, Spain; ^12^ Renal Transplant Unit, Department of Internal Medicine, Amsterdam University Medical Centers, Academic Medical Center—University of Amsterdam, Amsterdam, Netherlands; ^13^ Department of Clinical Sciences, Barcelona University, Barcelona, Spain

**Keywords:** kidney transplantation, calcineurin inhibitors immunosuppression, acute rejection, immunobiology, genetics

## Abstract

Achieving fast immunosuppression blood exposure after kidney transplantation is key to abrogating both preformed and *de novo* anti-donor humoral and cellular alloresponses. However, while tacrolimus (TAC) is the cornerstone immunosuppressant inhibiting adaptive alloimmunity, its blood exposure is directly impacted by different single-nucleotide polymorphisms (SNPs) in CYP3A TAC-metabolizing enzymes. Here, we investigated how functional TAC-CYP3A genetic variants (CYP3A4*22/CYP3A5*3) influence the main baseline clinical and immunological risk factors of biopsy-proven acute rejection (BPAR) by means of preformed donor-specific antibodies (DSAs) and donor-specific alloreactive T cells (DSTs) in a large European cohort of 447 kidney transplants receiving TAC-based immunosuppression. A total of 70 (15.7%) patients developed BPAR. Preformed DSAs and DSTs were observed in 12 (2.7%) and 227 (50.8%) patients, respectively. According to the different CYP3A4*22 and CYP3A5*3 functional allele variants, we found 4 differential new clusters impacting fasting TAC exposure after transplantation; 7 (1.6%) were classified as high metabolizers 1 (HM1), 71 (15.9%) as HM2, 324 (72.5%) as intermediate (IM), and 45 (10.1%) as poor metabolizers (PM1). HM1/2 showed significantly lower TAC trough levels and higher dose requirements than IM and PM (p < 0.001) and more frequently showed TAC underexposure (<5 ng/ml). Multivariate Cox regression analyses revealed that CYP3A HM1 and IM pharmacogenetic phenotypes (hazard ratio (HR) 12.566, 95% CI 1.99–79.36, p = 0.007, and HR 4.532, 95% CI 1.10–18.60, p = 0.036, respectively), preformed DSTs (HR 3.482, 95% CI 1.99–6.08, p < 0.001), DSAs (HR 4.421, 95% CI 1.63–11.98, p = 0.003), and delayed graft function (DGF) (HR 2.023, 95% CI 1.22–3.36, p = 0.006) independently predicted BPAR. Notably, a significant interaction between T-cell depletion and TAC underexposure was observed, showing a reduction of the BPAR risk (HR 0.264, 95% CI 0.08–0.92, p = 0.037). Such variables except for DSAs displayed a higher predictive risk for the development of T cell-mediated rejection (TCMR). Refinement of pretransplant monitoring by incorporating TAC CYP3A SNPs with preformed DSAs as well as DSTs may improve current rejection-risk stratification and help induction treatment decision-making.

## 1 Introduction

Alloreactive immune memory is the hallmark of adaptive immunity and is a key factor driving acute kidney transplant rejection and accelerated graft loss (1–3). Indeed, preformed donor-specific antibodies (DSAs) are a well-recognized factor of poor graft outcome, and owing to systematic pretransplant screening, the incidence of acute antibody-mediated rejection (ABMR) has significantly decreased (4). Likewise, preformed donor-specific T-cell memory (DSTs) may also exist in a great proportion of transplant candidates and has been associated with a higher risk of T cell-mediated rejection (TCMR) (5–8) after transplantation.

Importantly, memory T cells are more resistant to immunosuppressive therapies than their naïve counterparts (9–11), as they can rapidly repopulate and dominate peripheral anti-donor alloimmune responses (12). Experimental and human *ex vivo* studies have shown that calcineurin inhibitors, and especially tacrolimus (TAC), can more efficiently inhibit these cells (13, 14). However, even though the implementation of TAC-based regimens as the current standard of care immunosuppressive therapy has led to a significant reduction in acute rejection rates, acute TCMR still unpredictably occur (15, 16).

TAC has a narrow therapeutic index leading to a large interindividual pharmacokinetic variability (17), and suboptimal TAC exposure during the initial period after transplantation has been associated with a higher risk of acute rejection (18, 19), especially in highly immunized kidney transplant patients (20). Among different factors influencing TAC pharmacokinetics, single-nucleotide polymorphisms (SNPs) in genes coding for TAC-metabolizing enzymes cytochrome P450 (CYP) 3A4 and 3A5 have been shown to play a major impact (21, 22). Indeed, patients expressing the CYP3A5*1 allele (CYP3A5 expressers) have significantly higher dose requirements to achieve similar TAC trough levels (C_0_) than patients homozygous for the CYP3A5*3 allele (CYP3A5 non-expressers) (23, 24). Similarly, the non-functional CYP3A4*22 allele has also been associated with a reduced TAC dose requirement, regardless of CYP3A5 genotype (25, 26). Nevertheless, while genotype-based adjustment of initial TAC doses has proven useful in two prospective trials, no improvement on main clinical outcomes such as acute rejection rates has been described yet (27, 28). Of note, these studies did not stratify kidney transplant patients according to pretransplant alloimmune memory status, both DSAs and also DSTs, in whom different individual CYP3A TAC phenotype expression could modulate their risk of biopsy-proven acute rejection (BPAR).

Therefore, since kidney transplant candidates with preformed anti-donor alloimmune memory might need a particularly fast exposure to TAC blood concentrations to effectively inhibit anti-donor recall immune responses, particularly in the early posttransplant period, we hypothesized that the impact of pretransplant DSTs and DSAs, together with other main baseline clinical variables and the different CYP3A TAC phenotypes, could significantly modulate the relative risk and types of BPAR. Thus, the primary endpoint of the study was to evaluate the value of preformed alloimmune memory (both DSAs and DSTs) together with different CYP3A TAC pharmacogenetic phenotypes to discriminate patients at risk of developing acute rejection after kidney transplantation.

## 2 Materials and Methods

### 2.1 Study Population

**Figure 1:**
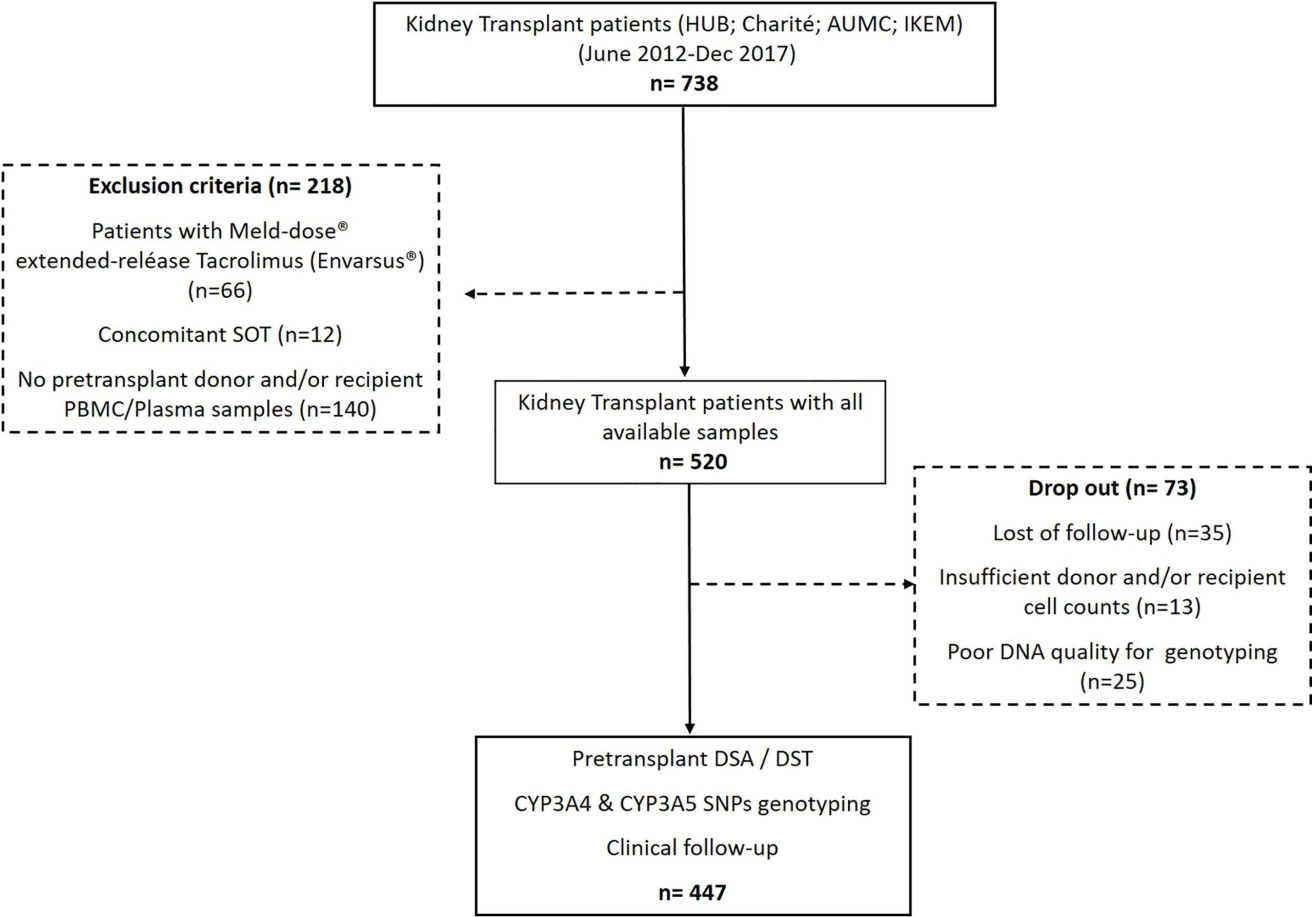
Flowchart of the study.

A total of 738 consecutive, adult, single-kidney-transplant recipients from four different European kidney transplant centers (Bellvitge University Hospital in Barcelona, Spain; Campus Virchow-Clinic in the Charité University Hospital in Berlin, Germany; Academic Medical Center, University of Amsterdam in Amsterdam, the Netherlands; and Institute for Clinical and Experimental Medicine (IKEM) in Prague, Czech Republic), who were transplanted between June 2012 and December 2017, were retrospectively analyzed on the basis of the availability of both donor and recipient pretransplant peripheral blood mononuclear cells (PBMCs) and recipient plasma samples to assess DSTs, DSAs, and the CYP3A genotypes for their value predicting acute rejection after transplantation. Furthermore, the main baseline and clinical variables such as the use of T cell-depleting agents, development of delayed graft function (DGF), and importantly distinct TAC blood exposures were also assessed in this study to evaluate their impact on modulating these pretransplant immunologic and pharmacogenetic variables facilitating the risk of acute rejection. As illustrated in **Figure 1**, 218 patients were excluded because they are receiving the Meld-dose® extended-release TAC formulation (Envarsus®), were transplanted with another concomitant solid organ, or lack of biological samples. The first exclusion criterion was applied due to the different pharmacokinetic profiles that have been reported for the Envarsus® formulation. In addition, 73 out of the 520 patients in the study were dropped out because of the following reasons: poor quality of DNA for genotyping analyses (n = 25), insufficient donor and/or recipient cell counts (n = 13), or lost to follow-up (n = 35). Therefore, 447 patients were evaluated in the study. The respective institutional review boards approved the study, and all patients gave written informed consent. Patients were followed up for at least 24 months. The main baseline demographic variables were collected at the time of enrollment, and clinical variables associated with clinical transplant outcomes were pooled together for the study. DGF was considered as the absence of recovery of graft function requiring hemodialysis after transplant surgery.

### 2.2 Immunosuppression

All patients of the study received an immediate (Prograf® Astellas Pharma; or Adoport® Sandoz Pharma) or extended-release (Advagraf®, Astellas Pharma) TAC formulation, mycophenolate mofetil (MMF), 1 g bid, during the first 2 weeks and subsequently tapered to 500 mg bid, and steroids with oral prednisone at 5 mg/day, after the first month as maintenance immunosuppression. Either basiliximab (72.3%) or thymoglobulin (rATG) (27.7%) was given as induction therapy as per practice in each center. Initial TAC doses were adjusted by the respective patient’s body weight and given at 0.05 mg/kg bid for the immediate release (TAC-IR) and 0.12 mg/kg/day for the extended release (TAC-ER), to achieve TAC trough levels of 6–10 ng/ml during the first 6 months and 5–8 ng/ml thereafter. TAC trough levels were measured before the patient’s administration of the morning dose, on days 7, 14, 30, and 90 and at 180 days after transplantation. TAC intra-patient variability (IPV) was estimated through the coefficient of variation (SD/mean × 100).

### 2.3 *CYP3A* Genotyping Analysis

Genomic DNA was extracted from a peripheral whole-blood sample using the Wizard Genomic DNA Purification Kit (Promega Corporation, Sydney, VIC, Australia) and was stored at −80°C. For the genotyping, allelic discrimination reactions were performed using specific TaqMan (Applied Biosystems, Foster City, CA, USA) genotyping assays on an ABI PRISM 7900 Fast Real-Time PCR Systems (Applied Biosystems) using 20 ng of genomic DNA and according to the manufacturer’s instructions. According to the functional CYP3A variants, patients were classified as poor TAC metabolizers (PM) if they were *22 carriers (*1/*22 or *22/*22) for CYP3A4 or they were *3/*3 genotype for CYP3A5 or as high metabolizers (HM) if they expressed the *1/*1 genotype for CYP3A4 or *1 carrier (*1/*3 or *1/*1) for CYP3A5. Furthermore, patients were clustered into different groups according to both CYP3A4*22 and CYP3A5*3 allelic status, specifically considering the functional *1 allele: high CYP3A metabolizers 1 (HM1) (CYP3A4*1/*1 and CYP3A5*1/*1), high metabolizers 2 (HM2) (either CYP3A4*1/*1 and CYP3A5*1 carriers or CYP3A4*1 carriers and CYP3A5*1/*1), intermediate (IM) (CYP3A4*1/*1 with CYP3A5*3/*3 or CYP3A4*22/*22 with CYP3A5*1/*1 or CYP3A4*22 carriers with CYP3A5*1 carriers), poor 1 (PM1) (CYP3A4*22 carriers with CYP3A5*3/*3 or CYP3A4*22/*22 with CYP3A5*1 carriers), and poor 2 (PM2) (CYP3A4*22/*22 with CYP3A5*3/*3). In the study cohort, there were no CYP3A4*22/*22 with CYP3A5*1/*1 patients (one of the combinations for IM) nor CYP3A4*22/*22 with CYP3A5*3/*3 (PM2).

### 2.4 Donor and Recipient Cell Source

Peripheral blood samples were obtained in heparinized tubes from renal transplant recipients before kidney transplantation. Donor cells were obtained from donor spleens or PBMCs in deceased and living donors. PBMCs and splenocytes were isolated by standard Ficoll density gradient centrifugation, were frozen in liquid nitrogen, and subsequently used for the IFN-γ Enzyme-linked Immunosorbent Spot (ELISPOT) assay.

### 2.5 Assessment of Pretransplant Donor-Specific Alloimmune Memory

Pretransplant humoral and cellular donor-specific alloimmune memory was assessed in all patients of the study in peripheral blood by means of serum DSAs and circulating donor-specific memory/effector T cells (DSTs), respectively. While pretransplant DSA data were available to the transplant physicians prior to transplantation, all data related to pretransplant DSTs were blinded and thus did not influence the type of immunosuppressive therapy used. Pretransplant DSAs were not detected at the time of transplantation and were confirmed later; therefore, these patients did not receive any desensitizing treatment before transplantation.

#### 2.5.1 Donor-Specific Anti-HLA Antibodies

Screening for circulating anti-HLA class I and II antibodies was carried out in serum samples before transplantation in all patients and at the time of a kidney transplant biopsy and was determined using single-antigen flow beads assays on a Luminex platform (Lifecodes, division of Immucor, Stanford, CT, USA). Patients previously only screened for anti-HLA antibodies with the screening assay (Lifecodes, division of Immucor, Stanford, CT, USA), were re-assessed for single-antigen flow beads assays to rule out the presence of donor (HLA)-specific antibodies (DSAs). All beads showing a normalized mean fluorescence intensity (MFI) >1,500 were considered positive if [MFI/(MFI lowest bead)] > 5.

#### 2.5.2 Donor-Specific Memory/Effector T Cells

Pretransplant frequencies of circulating donor-specific memory/effector T-cell alloreactivity (DSTs) were monitored by an IFN-γ ELISPOT assay following recently described standard operating procedures (29, 30). Briefly, 3 × 10^5^ responder cells were placed in triplicate wells with 3 × 10^5^ CD2-depleted splenocytes (Easysep® Human CD2 Selection kit, StemCell, Saint-Egrève, France) or CD3-depleted living-donor PBMCs (human CD3+ Cell Depletion Cocktail, RosetteSep® kit, StemCell, France). Recipient cells were stimulated with complete medium alone (RPMI 1640, GE Healthcare Life Sciences, Chicago, IL, USA, with 10% inactivated fetal bovine serum (FBS), antibiotics, and l-glutamine) and Pokeweed (AID, Autoimmun Diagnostika, Straßberg, Germany) as negative and positive controls, respectively. Results were given as frequencies of IFN-γ-producing donor-specific T cells/3 × 10^5^ PBMCs, subtracting responses of the negative control wells. As previously reported, 25 or higher IFN-γ-producing donor-specific T cells/3 × 10^5^ PBMCs was considered a positive test (4, 5).

### 2.6 Renal Allograft Histology

Renal allograft biopsies were performed in patients undergoing acute clinical graft dysfunction, such as a change in serum creatinine levels, decreasing estimated glomerular filtration rate (eGFR), and the appearance of proteinuria and/or hematuria. All renal biopsies were blindly analyzed following the Banff 2013 score classification (31) and retrospectively revised following Banff 2015 (32) by an expert kidney transplant pathologist.

### 2.7 Statistical Analysis

All data are presented as mean ± SD or median and interquartile range. Groups were compared using the X^2^-test for categorical variables and the one-way ANOVA analysis or Student’s t-test for normally distributed data for quantitative variables.

Cox regression analyses were performed to determine the significant univariate associations of pretransplantation factors with the risk of BPAR. An interaction analysis between ATG induction and low TAC exposure was also introduced, as it was suspected that a depleting ATG induction might dampen the effect of low TAC exposure. These interaction analyses were also carried out between ATG induction and DSTs, as well as ATG and DSAs. These interaction analyses were performed in Cox models with 2 covariates plus the interaction term. A multivariate Cox survival model was then built on these significant associations to evaluate the independent predictors of BPAR. Results were expressed as hazard ratios (HRs) with 95% CIs. Kaplan–Meier analyses were performed to represent allograft rejection-free survival, and log-rank tests were computed for the associated curves. The statistical significance level was defined as a 2-tailed p < 0.05. All statistical analyses were performed with IBM® SPSS Statistics (version 23) and R (version 4.1).

## 3 Results

### 3.1 Main Demographics and Clinical Characteristics of the Study Population

The flowchart of the study is depicted in **Figure 1**. The mean study patient follow-up was 36.4 ± 18.1 months. As shown in **Table 1**, most patients were adult Caucasian patients who underwent a deceased-donor kidney transplant. While only 12 patients showed pretransplant DSAs (2.7%), 227/447 (50.8%) displayed preformed DSTs. Most patients received a TAC-IR formulation (87.2%). A total of 323 (72.3%) patients received basiliximab, whereas 124 (27.7%) received rATG. Of 447, 70 (15.6%) developed BPAR, with most of them TCMR (57/70, 81.4%). The median time to BPAR was 0.95 [0.3–3.0] months. Thirteen (2.9%) patients died, and 33 (7.4%) allografts were lost.

**Table 1:** Main demographics of patients of the study.

Demographical, clinical, Immunological variables	Overall (n=447)
**Recipient age, yr**	51.450 (14.142)
**Donor age, yr**	54.492 (14.625)
**Recipient gender, female**	147 (32.9%)
**Donor type, living**	170 (38.0%)
**Recipient ethnicity, non-caucasian**	27 (6.0%)
**Transplant index, >1**	44 (9.7%)
**Time on dialysis, mo**	31.459 (39.622)
**Cold ischemia time, hr**	11.849 (9.389)
**Tacrolimus formulation**	
Immediate release (TAC-IR) Extended release (TAC-ER)	390 (87.2%) 57 (12.8%)
**rATG induction**	124 (27.7%)
**CYP3A genotypes**	
3A4*1*1, 3A5*1*1 (HM1)	7 (1.6%)
3A4*1*1, 3A5*1*3 (HM2)	71 (15.9%)
3A4*1*1, 3A5*3*3 (IM1)	319 (71.4%)
3A4*22, 3A5*1*3 (IM2)	5 (1.1%)
3A4*22*22, 3A5*1*1 (IM3)	0 (0%)
3A4*1*22, 3A5*3*3 (PM1)	45 (10.1%)
3A4*22*22, 3A5*3*3 (PM2)	0 (0%)
**HLA mismatches (class 1), #**	2.574 (1.024)
**HLA mismatches (class 2), #**	1.155 (0.607)
**PreTR DST, yes**	227 (50.8%)
**PreTR DSA, yes**	12 (2.7%)
**DGF, yes**	131 (29.3%)
**BPAR, yes**	70 (15.7%)
**TCMR**	57 (12.8%)
**ABMR**	18 (4.0%)
**Graft loss, yes**	33 (7.4%)
**Death, yes**	13 (2.9%)

ABMR, antibody-mediated rejection; BPAR, biopsy-proven acute rejection; DGF, delayed graft function; DSA, donor-specific antibodies; DST: donor-specific T cells; mo, months; PreTR, pre-transplant; rATG, rabit anti-thymocyte globulin; TCMR, T cell mediated rejection; yr, years.

The “*” is the symbol that represents the different allelic variants. It represents alleles with altered functionality which may lead to profiles of increased or reduced drug metabolism. # means "number"

### 3.2 Pharmacogenetic CYP3A Phenotypes and Posttransplant Tacrolimus Exposure

Since the patients of this study were treated with either an immediate (TAC-IR) or an extended-release (TAC-ER) TAC formulation, we first compared whether TAC trough concentrations and dose ratios (C/D ratio) between the two groups were comparable between the two formulations. As shown in **Supplementary Figure 1**, no differences were observed between TAC-IR and TAC-ER formulations, and thus, all patients were analyzed together (p > 0.05 for all time points).

The frequencies of the CYP3A4*1/*1 and *1/*22 genotypes were 397 (88.8%) and 50 (11.2%), respectively. There were no CYP3A4*22/*22 homozygous patients. The CYP3A5*1/1*, *1/*3, and *3/*3 genotypes were observed in 7 (1.6%), 76 (17%), and 364 (81.4%) patients, respectively. As previously described (24), patients with CYP3A4*1/*1 genotype were classified as high TAC metabolizers (HM) whereas *22 expressers as poor metabolizers (PM). Likewise, patients with CYP3A5*1/1*and *1/*3 genotypes were considered as TAC high metabolizers (HM) while *3/*3 genotype as poor metabolizers (PM). Since clustering patients according to both CYP3A4*22 and CYP3A5*3 allelic status may identify their metabolic status (24), we also stratified patients according to all 4 different CYP3A phenotype clusters in our cohort: 7/447 (1.6%) patients were classified as high HM1, 71/447 (15.9%) as HM2, 319/447 (71.4%) as IM1 and 5/447 (1.1%) as IM2, and 45/447 (10.1%) as PM1 (**Table 1**). As previously reported among the Caucasian transplant population (33), most CYP3A4*22 carriers were non-expressers for CYP3A5 (45/50; 90%).

**Figure 2:**
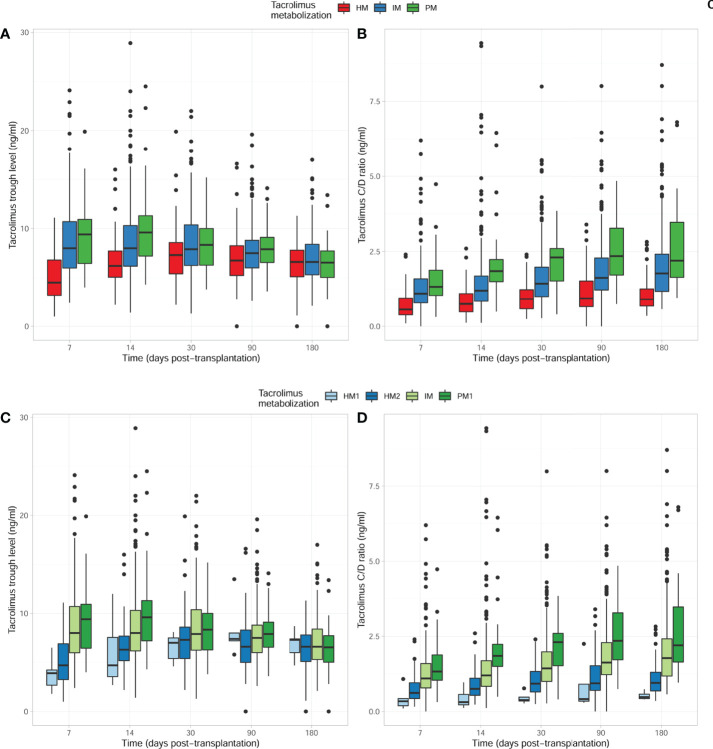
Comparison of tacrolimus outcomes with time, considering the four CYP3A TAC-metabolizing groups. **(A)** TAC trough levels between HM/IM and PM clusters. Significantly lower TAC levels were observed within the first 2 weeks early after transplantation in HM as compared to IM and PM patients. TAC C_0_ values (ng/ml) were 4.50 (3.15–6.85) vs 8.00 (5.97–10.70) vs 9.40 (6.40–11.05), p < 0.001 at 7 days; 6.20 (5.00–7.80) vs 8.00 (6.12–10.30) vs 9.60 (7.15–11.45), p < 0.001 at 14 days; 7.30 (5.37–8.60) vs 7.90 (6.20–10.39) vs 8.34 (6.22–10.31), p = 0.013 at 1 month; 6.75 (5.15–8.33) vs 7.50 (6.00–8.85) vs 7.90 (6.50–9.20), p = 0.029 at 3 months; and 6.60 (5.08–7.83) vs 6.60 (5.30–8.40) vs 6.53 (5.00–7.97), p = 0.517 at 6 months in HM CYP3A vs IM CYP3A vs PM CYP3A patients, respectively. **(B)** TAC trough level-to-dose ratio between HM/IM and PM clusters. HM patients required higher TAC doses to reach the same TAC trough levels as IM and PM at different times post-transplantation. TAC dose-adjusted C_0_ ratios were 0.56 (0.38–0.96) vs 1.10 (0.78–1.60) vs 1.32 (1.03–1.94), p < 0.001 at 7 days; 0.75 (0.47–1.10) vs 1.20 (0.83–1.69) vs 1.85 (1.50–2.30), p < 0.001 at 14 days; 0.92 (0.58–1.26) vs 1.43 (1.00–2.00) vs 2.30 (1.50–2.63), p < 0.001 at 1 month; 0.94 (0.64–1.55) vs 1.62 (1.22–2.29) vs 2.35 (1.66–3.30), p < 0.001 at 3 months; and 0.91 (0.68–1.28) vs 1.78 (1.18–2.43) vs 2.20 (1.62–3.50), p < 0.001 at 6 months in HM CYP3A vs IM CYP3A vs PM CYP3A patients, respectively. **(C)** TAC trough levels between HM1/2, IM, and PM1 clusters. Significantly lower TAC trough levels were observed within the first 2 weeks early after transplantation in HM1/2 as compared to IM and PM1 patients. TAC C_0_ values (ng/ml) were 3.90 (2.18–4.85) vs 4.70 (3.20–6.90) vs 8.00 (5.97–10.70) vs 9.40 (6.40–11.05), p < 0.001 at 7 days; 4.70 (2.90–8.30) vs 6.30 (5.13–7.76) vs 8.00 (6.12–10.30) vs 9.60 (7.15–11.45), p < 0.001 at 14 days; 7.00 (5.00–7.80) vs 7.30 (5.35–8.64) vs 7.90 (6.20–10.39) vs 8.34 (6.22–10.31), p = 0.028 at 1 month; 7.40 (6.50–10.75) vs 6.60 (4.95–8.35) vs 7.50 (6.00–8.85) vs 7.90 (6.50–9.20), p = 0.040 at 3 months; and 7.30 (5.35–8.05) vs 6.60 (5.05–7.85) vs 6.60 (5.30–8.40) vs 6.53 (5.00–7.97), p = 0.674 at 6 months in HM1 CYP3A vs HM2 CYP3A vs IM CYP3A vs PM1 CYP3A patients, respectively. **(D)** TAC trough level-to-dose ratio between HM1/2, IM, and PM1 clusters. HM1/2 patients required higher TAC doses to reach the same trough levels as IM and PM1 at different times post-transplantation. TAC dose-adjusted C_0_ ratios were 0.34 (0.14–0.60) vs 0.62 (0.41–0.96) vs 1.10 (0.78–1.60) vs 1.32 (1.03–1.94), p < 0.001 at 7 days; 0.31 (0.18–0.72) vs 0.76 (0.54–1.12) vs 1.20 (0.83–1.69) vs 1.85 (1.50–2.30), p < 0.001 at 14 days; 0.38 (0.30–0.67) vs 0.93 (0.64–1.40) vs 1.43 (1.00–2.00) vs 2.30 (1.50–2.63), p < 0.001 at 1 month; 0.41 (0.31–1.80) vs 0.94 (0.69–1.54) vs 1.62 (1.22–2.29) vs 2.35 (1.66–3.30), p < 0.001 at 3 months; and 0.48 (0.41–0.69) vs 0.95 (0.68–1.31) vs 1.78 (1.18–2.43) vs 2.20 (1.62–3.50), p < 0.001 at 6 months in HM1 CYP3A vs HM2 CYP3A vs IM CYP3A vs PM1 CYP3A patients, respectively. TAC, tacrolimus; HM, high metabolizers; IM, intermediate; PM, poor metabolizers.

When considering the CYP3A phenotypes’ categorization and their impact on first TAC trough levels and dose adjustments, we first analyzed the clusters by merging all HM, IM, and PM from each CYP3A genotype. As illustrated in **Figure 2**, HM patients showed significantly lower TAC trough levels as compared to IM and PM patients within the first 2 weeks. Notably, patients with an IM phenotype showed intermediate TAC trough levels compared to both HM and PM patients in the early period (**Figure 2A**). Also, HM required higher TAC dose adjustments to reach the same TAC trough levels than did PM during the first 6 months of follow-up (**Figure 2B**). This difference was also observed between IM and PM patients from 2 weeks to 6 months of follow-up. While TAC dose adjustments allowed TAC trough levels to converge starting 3 months post-transplantation, the C/D ratio remained significantly different between the three CYP3A cluster phenotypes. However, when the HM phenotype was further stratified into HM1 and HM2 phenotypes, statistically significant differences in both TAC trough levels and TAC dose adjustments (TAC C/D ratio) between the two HM groups were observed (**Figures 2C, D**). All pharmacokinetic data including TAC trough levels (ng/ml), daily doses (mg/day), and dose-adjusted trough level (ng/ml/mg/day) at five different time points (days 7 and 14 and months 1, 3, and 6) after transplantation are summarized in **Supplementary Table 1**.

### 3.3 Clinical and Demographic Baseline Characteristics of Different Pharmacogenetic CYP3A Clusters

As described in **Table 2**, there were no statistically significant differences between the different pharmacogenetic clusters regarding main baseline demographic, clinical, and immunological characteristics. Also, the type of induction therapy was not different between the groups. However, there was a higher number of non-Caucasian transplant recipients among the HM1 cluster than in IM and PM patients [3/7 (42.9%) non-Caucasian patients in HM1, 11/71 (15.5) in HM2, 10/324 (3.1%) in IM, and 3/45 (6.7%) in PM1; p < 0.001]. The mean time of dialysis duration was lower among PM1 as compared to HM1/2 and IM patients (p = 0.041). Also, a higher number of BPAR was observed among HM1/2 and IM as compared to PM [4/7 (57.1%) in HM1, 5/71 (7%) in HM2, 59/324 (18.2%) in IM, and 2/45 (4.4%) in PM patients; p < 0.001]. There were no observed differences between death-censored graft loss and patient survival.

**Table 2:** Main clinical, demographical and immunological characteristics between the four different TAC metabolizing groups.

	HM1 (n = 7)	HM2 (n = 71)	IM (n = 324)	PM1 (n = 45)	p value
**Clinical and demographic variables**					
**Recipient age, yr**	42.143 (17.620)	51.603 (13.385)	51.606 (14.529)	51.533 (11.673)	0.380
**Donor age, yr**	48.500 (15.398)	54.768 (13.892)	54.669 (14.793)	53.711 (14.815)	0.754
**Recipient gender, female**	1 (14.3%)	30 (42.3%)	104 (32.1%)	12 (26.7%)	0.187
**Donor type, living**	3 (42.9%)	24 (33.8%)	124 (38.3%)	19 (42.2%)	0.813
**Recipient ethnicity, non-Caucasian**	3 (42.9%)	11 (15.5%)	10 (3.1%)	3 (6.7%)	**<0.001**
**Previous transplants, #**	1.000 (0.000)	1.197 (0.435)	1.096 (0.361)	1.111 (0.318)	0.165
**Time on dialysis, mo**	34.229 (28.125)	38.609 (39.354)	31.833 (41.300)	16.953 (22.947)	**0.041**
**Cold ischemia time, hr**	11.602 (9.451)	12.046 (9.371)	11.891 (9.589)	11.308 (8.348)	0.982
**ATG induction**	2 (28.6%)	27 (38.0%)	85 (26.2%)	10 (22.2%)	0.187
**Immunological variables**					
**HLA mismatches (class 1), #**	2.333 (0.516)	2.406 (1.180)	2.597 (0.973)	2.705 (1.153)	0.381
**HLA mismatches (class 2), #**	1.500 (0.548)	1.130 (0.662)	1.147 (0.592)	1.205 (0.632)	0.495
**PreTR DSA**	1 (14.3%)	2 (2.8%)	8 (2.5%)	1 (2.2%)	0.295
**PreTR DST**	2 (28.6%)	33 (46.5%)	165 (50.9%)	27 (60.0%)	0.329
**Main clinical outcomes**					
**Delayed graft function**	2 (28.6%)	25 (35.2%)	94 (29.0%)	10 (22.2%)	0.512
**Rejection**					
**BPAR**	4 (57.1%)	5 (7.0%)	59 (18.2%)	2 (4.4%)	**<0.001**
**TCMR**	3 (42.9%)	5 (7.0%)	48 (14.8%)	1 (2.2%)	**0.004**
**ABMR**	2 (28.6%)	0 (0.0%)	15 (4.6%)	1 (2.2%)	**0.002**
**Mean time to BPAR**	3.975 (4.452)	1.874 (1.520)	1.976 (2.378)	0.550 (0.636)	0.357
**Graft loss**	1 (14.3%)	5 (7.0%)	25 (7.7%)	2 (4.4%)	0.772
**Death**	0 (0.0%)	3 (4.2%)	9 (2.8%)	1 (2.2%)	0.864

ABMR, antibody-mediated rejection; BPAR, biopsy-proven acute rejection; DGF, delayed graft function; DSA, donor-specific antibodies; DST: donor-specific T cells; HM, high metabolizer; mo, months; IM, intermediate metabolizer; PM, poor metabolizer; PreTR, pretransplant; rATG, rat anti-thymocyte globulin; TCMR, T cell mediated rejection; yr, years.

Bold values refer to p values of variables that are significantly different among the 4 different TAC metabolizing groups. # means "number".

### 3.4 CYP3A Clusters, Tacrolimus Trough Levels, and Biopsy-Proven Acute Rejection

**Figure 3:**
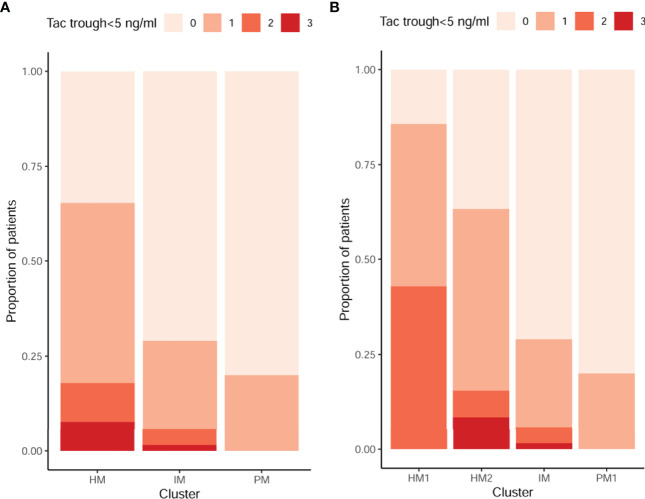
Proportion of TAC underexposure (<5 ng/ml) according to different CYP3A clusters. 0, 1, 2, and 3 in the legend represent the number of times that patients were off target. **(A)** There were a higher proportion of patients with TAC trough levels below 5 ng/ml among HM as compared to IM and PM at mean time of BPAR occurrence or before BPAR. The frequencies of patients with low levels at least once in this follow-up period were 20%, 29%, and 69% in the PM, IM, and HM groups, respectively (p < 0.001). **(B)** There were a higher proportion of patients with TAC trough levels below 5 ng/ml among HM1 and HM2 as compared to IM and PM1 at mean time of BPAR occurrence or before BPAR. The frequencies of patients with low levels at least once in this follow-up period were 20%, 29%, 63%, and 86% in the PM, IM, HM2, and HM1 groups, respectively (p < 0.001). TAC, tacrolimus; HM, high metabolizers; IM, intermediates; PM, poor metabolizers; BPAR, biopsy-proven acute rejection.

Median TAC trough levels were lower among patients developing BPAR as compared to those who did not at the mean time of BPAR occurrence (7.10 [5.90–9.01] vs 8.10 [6.47–9.74] ng/ml, respectively, p = 0.035). Among patients experiencing BPAR, 27/70 (38.6%) had at least one TAC trough level <5 ng/ml at any time prior to BPAR, whereas only 79/377 (21%) of patients without BPAR did (p = 0.001). This threshold was therefore considered as *low TAC exposure*. The proportion of patients with TAC trough levels below 5 ng/ml, at least once within the study follow-up, according to each CYP3A cluster phenotype was significantly higher among HM as compared to IM and PM (p < 0.001) (**Figure 3A**), as well as when HM was further stratified into HM1 and HM2 subgroups (p < 0.001) (**Figure 3B**).

### 3.5 Pretransplant Risk Factors Predicting Biopsy-Proven Acute Rejection

**Figure 4:**
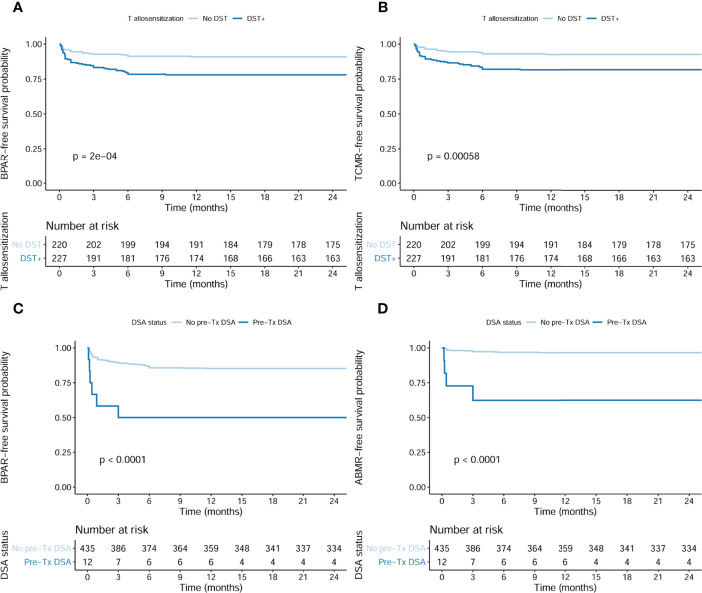
Incidence of BPAR in presence of pretransplant DSTs and DSAs in the whole population (n = 447). **(A)** Kaplan–Meier BPAR-free survival curves according to pretransplant DSTs (log rank <0.001). The incidence of BPAR was 50/227 (22%) in DST+ patients, whereas only 20/220 (9.1%) DST− developed BPAR, p < 0.001. **(B)** Kaplan–Meier TCMR-free survival curves according to pretransplant DSTs (log rank <0.001). The incidence of TCMR was 41/227 (18.1%) in DST+ patients, whereas only 16/220 (7.3%) DST− developed TCMR, p = 0.001. **(C)** Kaplan–Meier BPAR-free survival curves according to pretransplant DSAs (log rank <0.001). The incidence of BPAR was 6/12 (22%) in DSA+ patients, whereas only 64/435 (14.7%) DSA− developed BPAR, p = 0.001. **(D)** Kaplan–Meier ABMR-free survival curves according to pretransplant DSA (log rank <0.001). The incidence of ABMR was 4/12 (33.3%) in DSA+ patients, whereas only 14/435 (3.2%) DSA− developed ABMR, p < 0.001. BPAR, biopsy-proven acute rejection; DSTs, donor-specific alloreactive T cells; DSAs, donor-specific antibodies; TCMR, T cell-mediated rejection; ABMR, acute antibody-mediated rejection.

As illustrated in **Figure 4**, the presence of preformed DSTs or DSAs was associated with a higher incidence of BPAR (log rank <0.001 and log rank <0.001, respectively), especially with TCMR and ABMR, respectively (log rank <0.001 and log rank <0.001, respectively), although preformed DSTs did also associate with higher ABMR rates (6.2% in DST+ vs 1.8% in DST−, ABMR p = 0.019).

**Figure 5:**
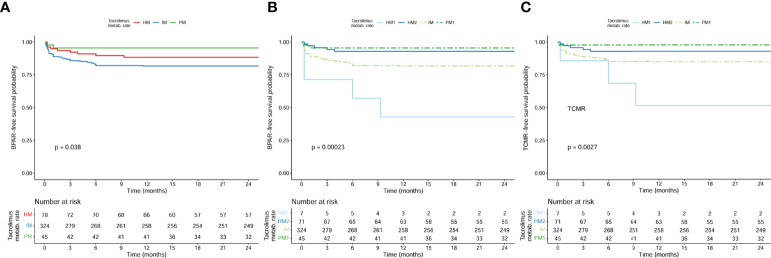
BPAR-free survival curves according to main CYP3A phenotype clusters (HM; IM and PM) (log rank = 0.038) and BPAR-free and TCMR-free survival curves according to the stratified clusters (HM1, HM2, IM, and PM1) (log rank <0.001 and log rank <0.001, respectively) in the whole population (n = 447). **(A)** Log rank (HM vs IM) = 0.156; log rank (HM vs PM) = 0.202; log rank (IM vs PM) = 0.026. **(B)** Log rank (HM1 vs HM2) < 0.001; log rank (HM1 vs IM) = 0.007; log rank (HM1 vs PM1) < 0.001; log rank (HM2 vs IM) = 0.023; log rank (HM2 vs PM1) = 0.593; log rank (IM vs PM1) = 0.026. **(C)** Log rank (HM1 vs HM2) < 0.001; log rank (HM1 vs IM) = 0.031; log rank (HM1 vs PM1) < 0.001; log rank (HM2 vs IM) = 0.077; log rank (HM2 vs PM1) = 0.274; log rank (IM vs PM1) = 0.024. BPAR, biopsy-proven acute rejection; HM, high metabolizers; IM, intermediate; PM, poor metabolizers; TCMR, T cell-mediated rejection.

When we analyzed BPAR-free survival curves according to main CYP3A phenotype clusters (HM; IM and PM), only IM patients showed a significantly lower cumulative incidence of BPAR than the HM and PM (**Figure 5A**) (log rank p = 0.038). Nevertheless, when we further stratified the HM cluster phenotype into the two HM1 and HM2 subgroups, patients displaying pharmacogenetic CYP3A clusters HM1 and IM showed significantly lower BPAR-free survival rates as compared to HM2 and PM1 patients (**Figure 5B**) (log rank p < 0.001). However, when TCMR-free survival rates were assessed (**Figure 5C**), only the HM1 group showed a significantly higher TCMR risk as compared to the other groups (p = 0.006 for HM1, p > 0.05 for IM and HM2, when compared to PM in a univariate Cox model).

Next, we assessed whether other relevant clinical or immunological variables were associated with the risk of BPAR, in univariate Cox analyses. As shown in **Table 3**, in addition to CYP3A clusters H1 and IM together with pretransplant DSTs and DSAs, the development of DGF and low TAC exposure (<5 ng/ml) were also correlates of BPAR. Conversely, while previous kidney transplantation, donor age, donor type (living vs brain dead), cold ischemia time, number of HLA mismatches, and rATG induction were not associated with BPAR, when we considered the combined effect of rATG induction and low TAC exposure, their interaction was associated with a significant reduction of BPAR (HR = 0.25, p = 0.025). Notably, when these significant covariates were assessed in a multivariate Cox regression analysis, CYP3A clusters (HM1 and IM), both preformed DSAs and DSTs, and DGF independently predicted BPAR. Although not statistically significant, a low TAC exposure (<5 ng/ml) showed a trend toward an increased risk of BPAR. Notably, the interaction between the use of rATG and TAC underexposure showed a significant reduction of the risk BPAR (HR = 0.312, p = 0.037). In other models adjusting for the interaction between rATG and DSTs or DSAs, while no significant interaction was observed, the risk of TCMR was numerically reduced in rATG-treated patients with DSTs but numerically increased in rATG patients with DSAs. Interestingly, when specifically focusing on the risk of TCMR, DSTs, DGF, recipient age, and the CYP3A HM1 phenotype as well as the interaction between rATG and low TAC exposure were associated with higher TCMR rates. While the interaction between DSTs and rATG induction was not significant (HR = 0.51, p = 0.308), it showed a numerically protective trend; i.e., patients with DSTs receiving rATG induction had a non-significantly lower risk of TCMR. Finally, in a multivariate Cox model, the three covariates DSTs, DGF, and recipient age remained independent predictors of the risk of TCMR; only the CYP3A HM1 phenotype, low TAC exposure, and the interaction between rATG and low TAC exposure were also independent correlates predicting TCMR.

**Table 3:** Univariate and Multivariate Cox analyses for clinical and immunological variables associated with the risk of BPAR.

	Univariate		Multivariate			
BPAR	HR	p-value	HR	95% CI	95% CI	p-value
**Cold ischemia time > 13 hr**	1.036	0.886				
**Recipient ethnicity**	0.968	0.949				
**Donor age > 65 yr**	0.924	0.787				
**Donor type (living)**	0.776	0.318				
**Kidney transplant index**	1.464	0.285				
**HLA MM (0 to 6. A, B, DR and DQ)**	1.11	0.273				
**PM1**	REF		REF			
**HM1**	15.978	**0.001**	12.566	1.99	79.348	**0.007**
**HM2**	1.51	0.590	1.398	0.266	7.357	0.692
**IM**	4.33	**0.042**	4.532	1.104	18.599	**0.036**
**DST**	2.58	**<0.001**	3.482	1.996	6.076	**<0.001**
**DSA**	4.796	**<0.001**	4.421	1.632	11.977	**0.003**
**DGF**	1.732	**0.024**	2.023	1.22	3.355	**0.006**
**ATG induction^1^**	1.761	0.072	1.477	0.779	2.799	0.232
**Low TAC exposure^1,2^**	1.83	**0.035**	1.619	0.889	2.952	0.115
**TAC IPV**	1.005	0.45				
**Interaction^1^ ATG / low TAC exp**	0.247	**0.025**	0.264	0.076	0.92	**0.037**
**Interaction^3^ ATG / DST**	0.597	0.360				
**Interaction^4^ ATG / DSA**	1.38	0.723				
TCMR	HR	p-value	HR	95% CI	95% CI	p-value
**Cold ischemia time > 13 hr**	0.99	0.886				
**Recipient ethnicity**	1.21	0.719				
**Recipient age**	0.98	0.046	0.98	0.96	1.0	0.050
**Donor age > 65 yr**	1.00	0.989				
**Donor type (living)**	0.73	0.271				
**Kidney transplant index**	0.90	0.813				
**HLA MM (0 to 6. A, B, DR and DQ)**	1.06	0.598				
**PM1**	REF		REF			
**HM1**	23.84	**0.006**	20.69	2.01	213.54	**0.011**
**HM2**	3.15	0.296	2.61	0.29	22.81	0.387
**IM**	7.06	0.053	4.532	0.98	51.70	0.053
**DST**	2.65	**<0.001**	3.30	1.82	5.98	**<0.001**
**DSA**	1.87	0.386				
**DGF**	1.87	**0.018**	2.60	1.49	4.56	**<0.001**
**ATG induction^1^**	0.85	0.589	1.27	0.60	2.70	0.52
**Low TAC exposure^1,2^**	1.60	**0.076**	2.11	1.12	3.98	**0.021**
**IPV IPV**	1.001	0.845				
**Interaction^1^ ATG / low TAC exp**	0.13	**0.014**	0.14	0.03	0.70	**0.017**
**Interaction^3^ ATG / DST**	0.51	0.308				

BPAR, biopsy-proven acute rejection; DGF, delayed graft function; DSA, donor-specific antibodies; DST: donor-specific T cells; HM; high metabolizer; hr, hours; IM, intermediate metabolizer; IPV, intra-patient variability; PM, poor metabolizer; PreTR, pretransplant; rATG, rat anti-thymocyte globulin; TAC, Tacrolimus; TCMR, T cell mediated rejection; yr, years.

^1^Interaction in a two-way model with rATG and low TAC exposure as predictors, with their interaction.

^2^Low TAC exposure corresponds to any TAC trough measurement below 5 ng/ml prior to BPAR or until end of follow-up.

^3^Interaction in a two-way model with rATG and DST as predictors, with their interaction.

^4^Interaction in a two-way model with rATG and DSA as predictors, with their interaction.

Bold values to p values of variables that are significantly associated with the risk of BPAR or TCMR.

Since rATG induces a deep T-cell depletion and although this variable was included in the multivariate analysis, we also performed a BPAR-free Cox survival analysis restricted to patients not receiving rATG induction. In this analysis, preformed DSTs, CYP3A cluster HM1, DGF, and low TAC exposure remained independently associated with BPAR (**Supplementary Table 2**).

Finally, given that two different BPAR density peaks were identified over time, *post-hoc* analyses were performed to compare the two subpopulations of early (<4 months) and late (>4 months) BPAR (**Supplementary Table 3**). Notably, the same independent predictive variables described when the whole study population was analyzed together were also confirmed when stratifying by either early or late BPAR (data not shown).

## 4 Discussion

This is the first study in solid organ transplant recipients evaluating the impact of main TAC CYP3A pharmacogenetic variants together with main immunologic biomarkers tracking preformed anti-donor alloimmune memory predicting the risk of posttransplant acute rejection. In this large, multicentric, European kidney transplant cohort, we first describe a further refined stratification of CYP3A pharmacogenetic phenotype clusters from those three previously described in the literature (22), which are significantly associated with different TAC metabolizer profiles. Indeed, this new categorization identifies kidney transplant recipients with distinct first fast TAC trough levels and C/D ratios and discriminates those patients at higher risk of both BPAR and TCMR, which was also directly influenced by low previous TAC exposure. Furthermore, we confirm the persistent independent deleterious effects of preformed DSAs and DSTs on the risk of BPAR, independent of these distinct TAC-metabolizing phenotypes. Most notably, a significant protective effect on the risk of BPAR was observed with the use of T cell-depleting agents such as rATG in the setting of TAC underexposure.

In the last years, an important body of evidence has shown the relevance of specific SNPs of the two main variants of the CYP3A TAC-metabolism enzymes (CYP3A4*22 and CYP3A5*3), leading to distinct functional phenotypes influencing TAC dose requirements to achieve whole blood pre-dose concentrations (C_0_) in kidney transplant recipients (21–26). In line with previous reports, we here show the impact of the two main CYP3A SNPs, on both TAC C_0_ and TAC dose requirements; indeed, HM1/2 had a significantly lower TAC dose-adjusted C_0_ ratios than IM, and PM patients had significantly higher TAC dose-adjusted C_0_ ratios compared with IM patients. It is interesting to observe that while the prevalence of each gene variant is largely dependent on the ethnicity of the patients, in our study, mainly represented by Caucasian kidney transplant recipients, there was a small but significant proportion of CYP3A5 expressers and CYP3A4*22 allele carriers, which led to a representative number of patients with a distinct global TAC-metabolizing capacity.

Nevertheless, while genotype-based adjustment of TAC doses in the initial course of kidney transplantation has been shown to be useful to more accurately and rapidly reach the target C_0_ shortly after transplantation, no advantages have been demonstrated in terms of improved clinical outcomes when prospectively assessed (27, 28). Here, by using this new categorization considering the functional *1 allele, we observed that while CYP3A HM1 patients are at higher risk of BPAR and TCMR as compared to other clusters, PM transplant recipients seem to display a significantly lower BPAR risk as compared to other CYP3A clusters. Unexpectedly, the IM cluster showed a deleterious effect on the global BPAR risk over PM but also over the HM2 group. These findings might be explained by the high number of patients in the IM group, which is the most frequent in our population, thus inferring more heterogeneity among this group than the probably much more homogeneous HM1/2 and PM1 groups. Nonetheless, when evaluating their impact on TCMR only, now the HM1 phenotype was revealed the most relevant factor driving this type of rejection. Therefore, the study of TAC CYP3A pharmacogenetic variants should be encouraged, especially among study cohorts with a greater representation of non-Caucasian individuals.

Importantly, we confirm that patients developing BPAR were significantly underexposed to TAC than patients not developing BPAR. Indeed, the effect of early low TAC exposure (trough levels below 5 ng/ml) barely independently correlated to BPAR, thus underscoring that initial low TAC trough levels may facilitate anti-donor alloimmune activation triggering allograft rejection. Moreover, a significant interaction between rATG and low TAC exposure was observed, leading to a reduction in the risk of BPAR. This interaction, together with the finding of the independent predictive value of TAC subexposure among patients not receiving rATG, strongly suggests that T-cell depletion induction therapy mitigates the risk of early low TAC exposures, conferring a protective umbrella in patients with insufficient immunosuppressive coverage, with HM patients the most suitable group. Alternatively, earlier TAC initiation or higher TAC dosage could eventually also counterbalance this deleterious effect.

Another interesting finding in this work is that we confirm the independent negative effect of preformed DSTs and DSAs on the risk of BPAR. Previous studies by our group and others have reported a strong association between pretransplant DSTs and increased risk of BPAR, particularly TCMR, during the early period after transplantation, especially in patients not receiving T cell-depleting induction therapies (3, 18, 19). Indeed, induction protocols using rATG have marked lymphopenia effects resulting in subsequent induction of apoptosis and/or anergy (20). In line with these observations, although not significant, the interaction effect between rATG and DSTs showed a clinically relevant trend (HR < 1), reducing the risk of BPAR. To note, while no additional deleterious effect was observed within HM patients with preformed DSTs or DSAs as compared to those patients with an IM or PM phenotype, the low number of immunological events within each pharmacogenetic phenotype with preformed DSA or DST group may have precluded detecting statistical differences.

Our study has some limitations. Despite the high number of patients evaluated, the ethnicity of our study population, which is representative of most European kidney transplant programs, was mostly Caucasian; thus, certain CYP3A SNPs were less represented. Nevertheless, the similar distribution of main demographic, clinical, and immunological risk factors within all CYP3A pharmacogenetic clusters significantly counterbalance this constraint. We also acknowledge that there are other relevant variables that may directly influence TAC pharmacokinetics variability in whole blood in addition to the CYP3A genotypes, such as patient hematocrit, weight, corticosteroid dose, and a reduction of the hepatic function. Nevertheless, the impact of the TAC-metabolizing CYP3A genotypes on TAC dose requirements to achieve whole blood pre-dose concentrations highlights the important effects of the individual genetic susceptibility on TAC blood exposure. We did not include subclinical rejections; thus, a number of additional immune-mediated events may have occurred in our study population and have not been taken into account. Finally, the short follow-up period of the study may not have allowed us to observe some additional deleterious impacts in the long term.

In conclusion, the results of our study strongly suggest that implementing pretransplant anti-donor alloimmune memory, both humoral and cellular, together with the individual genetic TAC-metabolizing susceptibility may significantly refine current immune-risk stratification of kidney transplant candidates prior to transplantation and may help in guiding treatment decision-making in a more personalized manner. Notably, these data warrant the development of large, prospective biomarker-guided trials, preferentially within multicenter international consortia.

## Data Availability Statement

The original contributions presented in the study are included in the article/**Supplementary Material**, further inquiries can be directed to the corresponding authors.

## Ethics Statement

The studies involving human participants were reviewed and approved by the Investigator Research Board of Bellvitge University Hospital. The patients/participants provided their written informed consent to participate in this study.

## Author Contributions

This article has been approved by all authors. NL and OB participated in the research design. EC, MS, SM, MM, and AT conducted the immune assay experiments. AV-A and PF performed the genotyping analysis. AS, MM, AF, JC, JG, FM, PR, and OB performed the clinical follow-up of patients of the study and the collection of clinical data. MG analyzed the histology. EC, AV-A, TJ, SM, LD, PH, OV, FB, NL, and OB performed the data collection and analysis. EC, TJ, NL, and OB wrote the article. PR, JG, NL, and OB developed refined study concepts, finalized the article draft, and secured the funding. All authors listed have made a substantial, direct, and intellectual contribution to the work and approved it for publication.

## Funding

This work was supported by the Instituto de Salud Carlos III (ISCIII) (grant numbers PI16/01321, PI19/01710, and PI18/P1740) (co-funded by European Regional Development Fund, ERDF, a way to build Europe). Also, this work was partly supported by the SLT002/16/00183 grant, from the Department of Health of the Generalitat de Catalunya by the call “Acció instrumental de programes de recerca orientats en l’àmbit de la recerca i la innovació en salut.” The authors thank the Research Centers of Catalonia (CERCA) Programme/Generalitat de Catalunya for institutional support. OB was awarded an intensification grant from the “Instituto de Salud Carlos III” [NT19/00051].

## Conflict of Interest

The authors declare that the research was conducted in the absence of any commercial or financial relationships that could be construed as a potential conflict of interest.

The reviewer L.R has declared a shared affiliation with the author T.J to the handling editor at the time of review.

## Publisher’s Note

All claims expressed in this article are solely those of the authors and do not necessarily represent those of their affiliated organizations, or those of the publisher, the editors and the reviewers. Any product that may be evaluated in this article, or claim that may be made by its manufacturer, is not guaranteed or endorsed by the publisher.
