# Supplementary material for: Tacrolimus CYP3A Single-Nucleotide Polymorphisms and Preformed T- and B-Cell Alloimmune Memory Improve Current Pretransplant Rejection-Risk Stratification in Kidney Transplantation
Source: Front Immunol. 2022 Jun 27;13:869554. doi: 10.3389/fimmu.2022.869554 (PMC9272702; doi:10.3389/fimmu.2022.869554)
Supplement: Supplementary file 1 [file DataSheet_1.zip › Supplementary Material.docx]

**Supplemental Figure 1**: dose-adjusted C_0_ at day 7, day 14, months 1, 3 and 6 after transplantation in patients receiving either immediate-release TAC formulation (TAC-IR) or extended-release TAC formulation (TAC-ER).


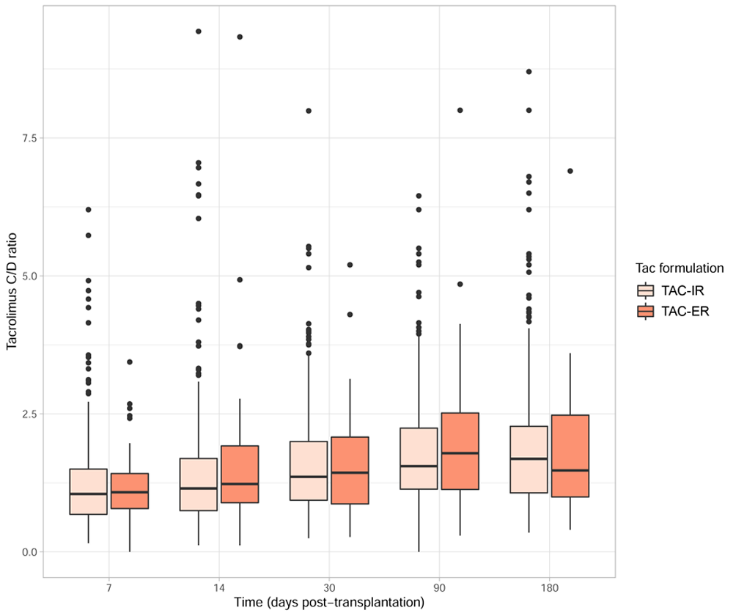


**Supplemental Table 1:** Tacrolimus trough levels (C_0_), daily dose and dose-adjusted C_0_ at day 7, day 14, months 1, 3 and 6 after transplantation in the study population considering the CYP3A cluster phenotype.

|  | **High Metabolizer (HM1)**  **n=7 (1.6%)** | **High Metabolizer (HM2)**  **n=71 (15.9%)** | **Intermediate Metabolizer**  **(IM)**  **n=324 (72.5%)** | **Poor Metabolizer (PM1)**  **n=45 (10.1%)** | **p value** |
| --- | --- | --- | --- | --- | --- |
| C_0_ (ng/mL) day 7 | 3.78 (1.66) | 5.15 (2.33) | 8.71 (3.87) | 9.18 (3.63) | **< 0.001** |
| C_0_ (ng/mL) day 14 | 5.94 (3.35) | 6.66 (2.62) | 8.55 (3.68) | 10.07 (4.24) | **< 0.001** |
| C_0_ (ng/mL) month 1 | 6.52 (1.47) | 7.37 (2.95) | 8.40 (3.31) | 8.67 (2.94) | **0.045** |
| C_0_ (ng/mL) month 3 | 8.38 (2.97) | 6.85 (2.73) | 7.67 (2.59) | 7.94 (2.33) | 0.071 |
| C_0_ (ng/mL) month 6 | 6.82 (1.52) | 6.37 (2.41) | 6.94 (2.45) | 6.58 (2.47) | 0.397 |
| Dose (mg/day) day 7 | 12.67 (6.92) | 8.19 (4.84) | 7.66 (3.21) | 6.96 (3.03) | **0.003** |
| Dose (mg/day) day 14 | 14.83 (7.03) | 8.85 (5.21) | 7.26 (3.86) | 5.64 (2.56) | **< 0.001** |
| Dose (mg/day) month 1 | 16.75 (7.18) | 8.39 (5.21) | 6.25 (3.45) | 4.56 (2.15) | **< 0.001** |
| Dose (mg/day) month 3 | 15.50 (6.61) | 7.31 (4.29) | 4.84 (2.52) | 3.74 (2.00) | **< 0.001** |
| Dose (mg/day) month 6 | 14.25 (5.56) | 6.89 (3.12) | 4.20 (2.10) | 2.99 (1.29) | **< 0.001** |
| Dose-adjusted C_0_ (ng/mL/mg/kg/day) day 7 | 0.41 (0.35) | 0.76 (0.50) | 1.32 (0.87) | 1.56 (0.88) | **< 0.001** |
| Dose-adjusted C_0_ (ng/mL/mg/kg/day) day 14 | 0.43 (0.32) | 0.91 (0.55) | 1.49 (1.19) | 2.07 (1.25) | **< 0.001** |
| Dose-adjusted C_0_ (ng/mL/mg/kg/day) month 1 | 0.45 (0.22) | 1.05 (0.57) | 1.67 (1.06) | 2.11 (0.79) | **< 0.001** |
| Dose-adjusted C_0_ (ng/mL/mg/kg/day) month 3 | 0.84 (0.94) | 1.13 (0.68) | 1.90 (1.07) | 2.48 (1.01) | **< 0.001** |
| Dose-adjusted C_0_ (ng/mL/mg/kg/day) month 6 | 0.52 (0.15) | 1.12 (0.61) | 2.02 (1.22) | 2.64 (1.47) | **< 0.001** |

**Supplemental Table 2:** Univariate and Multivariate Cox analyses for clinical and immunological variables associated with the risk of BPAR in patients not receiving T cell depleting agents.

|  | **Univariate** | | **Multivariate** | | | |  |
| --- | --- | --- | --- | --- | --- | --- | --- |
| **BPAR** | **HR** | **p-value** | **HR** | **95% CI** | **95% CI** | **p-value** |  |
| **Cold ischemia time > 13 hr** | 1.29 | 0.397 |  |  |  |  |  |
| **Recipient ethnicity** | 1.54 | 0.406 |  |  |  |  |  |
| **Donor age > 65 yr** | 1.02 | 0.963 |  |  |  |  |  |
| **Donor type (living)** | 0.61 | 0.113 |  |  |  |  |  |
| **Kidney transplant index** | 2.24 | 0.088 |  |  |  |  |  |
| **HLA MM (0 to 6. A, B, DR and DQ)** | 1.14 | 0.270 |  |  |  |  |  |
| **PM1** | REF |  | REF |  |  |  |  |
| **HM1** | 11.84 | **0.007** | 10.81 | 1.43 | 81.70 | **0.021** |  |
| **HM2** | 2.02 | 0.401 | 1.54 | 0.29 | 8.34 | 0.614 |  |
| **IM** | 3.00 | **0.129** | 3.21 | 0.77 | 13.36 | **0.109** |  |
| **DST** | 3.00 | **<0.001** | 3.96 | 2.01 | 7.79 | **<0.001** |  |
| **DSA** | 3.89 | **0.060** | 2.58 | 0.43 | 15.5 | **0.003** |  |
| **DGF** | 1.732 | **0.024** | 2.21 | 1.20 | 4.07 | **0.011** |  |
| **Low TAC exposure^1,2^** | 1.82 | **0.036** | 1.53 | 0.83 | 2.82 | 0.176 |  |
| **Abbreviations:** BPAR, biopsy-proven acute rejection; DGF, delayed graft function; DSA, donor-specific antibodies; DST: donor-specific T cells; HM, high metabolizer; hr, hours; IM, intermediate metabolizer; PM, poor metabolizer; PreTR, pre-transplant; rATG, rabit anti-thymocyte globulin; TAC, Tacrolimus; yr, years. | | | | | | | |

**Supplemental Table 3:** Main clinical, demographical and immunological characteristics between early and late BPAR.

|  | **Early BPAR (n=56)** | **Late BPAR (n=14)** | **p value** |
| --- | --- | --- | --- |
| **Recipient age, yr** | 48.85 (14.97) | 48.43 (14.97) | 0.926 |
| **Recipient gender, female** | 22 (39.3%) | 2 (14.3%) | 0.078 |
| **Recipient ethnicity, non Caucasian** | 4 (7.1%) | 0 (0.0%) | 0.303 |
| **Time on dialysis, mo** | 39.47 (36.68) | 40.45 (51.75) | 0.936 |
| **Donor age, yr** | 53.87 (13.25) | 50.43 (14.64) | 0.398 |
| **Donor type, living** | 19 (33.9%) | 4 (28.6%) | 0.703 |
| **HLA mismatches, # (0 to 6. A, B, DR and DQ)** | 3.93 (1.14) | 3.69 (0.95) | 0.493 |
| **Cluster** |  |  | 0.261 |
| **HM1** | 2 (3.6%) | 2 (14.3%) |  |
| **HM2** | 5 (8.9%) | 0 (0.0%) |  |
| **IM** | 47 (83.9%) | 12 (85.7%) |  |
| **PM1** | 2 (3.6%) | 0 (0.0%) |  |
| **PreTR DSA** | 6 (10.7%) | 0 (0.0%) | 0.200 |
| **PreTR DST** | 40 (71.4%) | 10 (71.4%) | 1.000 |
| **Low tac exposure, #** |  |  | 0.418 |
| **0** | 35 (62.5%) | 7 (50.0%) |  |
| **1** | 18 (32.1%) | 5 (35.7%) |  |
| **2** | 2 (3.6%) | 2 (14.3%) |  |
| **3** | 1 (1.8%) | 0 (0.0%) |  |
| **ATG induction** | 15 (26.8%) | 6 (42.9%) | 0.241 |
| **DGF** | 24 (42.9%) | 4 (28.6%) | 0.329 |
| **Abbreviations:** BPAR, biopsy-proven acute rejection; DGF, delayed graft function; DSA, donor-specific antibodies; DST: donor-specific T cells; PreTR, pre-transplant; rATG, rabit anti-thymocyte globulin; TAC, Tacrolimus; yr, years. | | | |
